# Endocannabinoid Levels in Ulcerative Colitis Patients Correlate With Clinical Parameters and Are Affected by Cannabis Consumption

**DOI:** 10.3389/fendo.2021.685289

**Published:** 2021-08-31

**Authors:** Shelly Tartakover Matalon, Shahar Azar, David Meiri, Rivka Hadar, Alina Nemirovski, Narjes Abu Jabal, Fred Meir Konikoff, Liat Drucker, Joseph Tam, Timna Naftali

**Affiliations:** ^1^Sackler School of Medicine, Tel Aviv University, Tel Aviv, Israel; ^2^Institute of Gastroenterology and Hepatology, Meir Medical Center, Kfar Saba, Israel; ^3^Obesity and Metabolism Laboratory, Institute for Drug Research, School of Pharmacy, Faculty of Medicine, The Hebrew University of Jerusalem, Jerusalem, Israel; ^4^Department of Biology, Technion-Israel Institute of Technology, Haifa, Israel

**Keywords:** cannabis, ulcerative colitis, Crohn’s disease, inflammatory bowel disease, endocannabinoids

## Abstract

**Background:**

Inflammatory bowel diseases (IBDs) are chronic, idiopathic, inflammatory, gastrointestinal disorders. The endocannabinoid system may have a role in the pathogenesis of IBD. We aimed to assess whether cannabis treatment influences endocannabinoids (eCBs) level and clinical symptoms of IBD patients.

**Methods:**

Blood samples and biopsies were taken from IBD patients treated by either cannabis or placebo for 8 weeks. Immunohistochemistry for *N*-acyl-phosphatidylethanolamine-selective phospholipase D (NAPE-PLD) and fatty acid amide hydrolase (FAAH) expression was done on colon biopsies, and sample levels of anandamide (AEA), eCB2-arachidonylglycerol (2-AG), arachidonic acid (AA), palmitoylethanolamine (PEA), and oleoylethanolamine (OEA) were measured in patient’s sera before and after cannabis treatment. Caco-2 cells were cultured with extracts of cannabis with/without tetrahydrocannabinol (THC) and their proteins extracted, and Western blotting for NAPE-PLD and FAAH expression was done.

**Results:**

Thirteen patients with Crohn’s disease (CD) and nine patients with ulcerative colitis (UC) were treated with cannabis. Seventeen patients with CD and 10 with UC served as placebo groups. In all CD patients, the levels of eCBs remained unaltered during the treatment period. In UC patients treated with placebo, but not in those treated with cannabis, the levels of PEA, AEA, and AA decreased significantly. The percent reduction in bowel movements was negatively correlated with changes observed in the circulating AEA and OEA, whereas improvement in quality of life was positively correlated with the levels of 2-AG. In the biopsies from UC patients, FAAH levels increased over the study period. In Caco-2 cells, both cannabis extracts increased NAPE-PLD levels but reduced FAAH expression levels.

**Conclusion:**

Our study supports the notion that cannabis use affects eCB “tone” in UC patients and may have beneficial effects on disease symptoms in UC patients.

## Introduction

Inflammatory bowel diseases (IBD), comprising Crohn’s disease (CD) and ulcerative colitis (UC), are chronic, idiopathic, inflammatory, gastrointestinal (GI) disorders. The pathogenesis of IBD is multifactorial and relates to dysregulated immune response to environmental factors in genetically susceptible hosts (1, 2). IBD poses a significant burden on patients because of detrimental effects on quality of life, growth, and development. Despite the introduction of new therapies, response to current treatments is limited to only 40%–60% (2, 3). Thus, new therapies for IBD with novel mechanisms of action are needed.

A common alternative is the therapeutic use of cannabis, reported by many IBD patients who claim that it improves pain, diarrhea, and appetite (4). Exogenous cannabinoids exert their influence through the endocannabinoid system (ECS). This system consists of cannabinoid receptors (CBRs: CB1, CB2, GPR55, TRPV1, and PPARs), endogenous cannabinoid ligands, endocannabinoids (eCBs), and their synthesizing and degrading enzymes (5). The eCBs are a family of bioactive lipids that are produced “on demand” in response to membrane depolarization and calcium influx and originate mainly from the hydrolysis of membrane phospholipids. The eCBs anandamide (AEA), oleoylethanolamine (OEA), and palmitoylethanolamine (PEA) are produced from *N*-acyl-phosphatidylethanolamine (NAPE) either directly through a NAPE-selective phospholipase D (NAPE-PLD) or through a serial action of lipases (6). The eCB2-arachidonylglycerol (2-AG) is produced from diacylglycerol by diacylglycerol lipase (DAGL) (7). AEA, PEA, and OEA are broken down by the enzyme fatty acid amide hydrolase (FAAH) to arachidonic acid (AA) and ethanolamine, whereas 2-AG is, to some extent, metabolized by FAAH but primarily broken down by the enzyme monoacylglycerol lipase (MAGL) to AA and glycerol.

The ECS does not appear to be tonically active under physiological conditions. However, numerous alterations in CBRs density and in eCBs level, inducing changes in the endocannabinoid tone, have been noted under various pathophysiological conditions (8). Previous studies have suggested that the ECS affects a variety of functions and events in the GI system (9, 10) and may have a role in the pathogenesis of IBD (5). Indeed, various reports showed altered regulation of the ECS in IBD patients, yet with rather contradictory outcomes (11). While controversies exist regarding the level of eCBs in IBD patients, some studies observed decreased activity and/or levels of the synthesizing enzyme NAPE-PLD and increased activity of the degrading enzyme FAAH (11).

Experimental studies in rodents (5) and recent clinical trials including from our group suggested that treatment with cannabinoids/cannabis may benefit patients with IBD (12–15). Previously, we conducted two double-blind, randomized, placebo-controlled trials. In the first one, UC patients received either cigarettes containing dried cannabis inflorescence with 23% tetrahydrocannabinol (THC) or placebo cigarettes without the THC for 8 weeks. Lichtiger disease activity index and quality of life (QOL) significantly improved in the cannabis group in comparison to the placebo group. In the second one, CD patients received cannabis oil containing 160/40 mg/ml cannabidiol (CBD)/THC or placebo for 8 weeks. Crohn’s disease activity index (CDAI) and QOL were significantly improved in the cannabis group compared to the placebo group.

In the current study, in order to shed some light on potential mechanisms of cannabis treatment in IBD, we assessed whether treating IBD patients with cannabis affects eCBs level and whether these changes correlate with clinical symptoms. To that end, we used sera collected in previous studies (14, 15). These sera were analyzed for eCBs level in UC and CD patients before and after 8 weeks of treatment with cannabis or placebo. We also assessed FAAH and NAPE-PLD levels in biopsies collected from the colon of UC patients before and after cannabis treatment. Finally, we determined the effect of cannabis on the protein levels of FAAH and NAPE-PLD in Caco-2 cells (colon carcinoma epithelial cells).

## Methods

### Study Population

#### UC Patients

The study population included patients (20–80 years old) with mild to moderate UC diagnosed at least 3 months prior to enrollment. Disease severity was determined by Lichtiger score of ≥4 to ≤15 and Mayo endoscopic subscore ≥1. The study was approved by the Meir Hospital ethical committee and the Israeli Ministry of Health, No. 0308-13-MMC.

#### CD Patients

Patients (age 20–80 years) with mild to moderate CD diagnosed at least 3 months prior to enrollment were included. Disease activity was determined by CD activity index of ≥200 and simple endoscopic score for CD (SES-CD) >2.The study was approved by the Meir Hospital ethical committee and the Israeli Ministry of Health, No. 0190-13MMC.

### Studies Protocols

Patients were allowed to continue their chronic IBD medications as long as they were on a stable dose. Specifically, at least 4 weeks for 5-aminosalicylates and at least 3 months for immunomodulators and biological treatments. Prednisone was permitted if patients were on a stable dose for at least 8 weeks prior to study onset. Patients were specifically asked to avoid any change in their medications during participation in the study. Exclusion criteria included the use of cannabis, whether for medical or recreational purposes, being pregnant or lactating, severe UC (Mayo score >10) or severe CD (CDAI >400), proctitis (i.e., inflammatory segment of <15 cm), known psychiatric diagnosis, or addiction traits based on self-reporting or noted in the patient electronic medical records. Patients who required an operation within the study period were excluded.

Patients were randomly assigned using a block method (16) in a 1:1 ratio to receive either medical cannabis or placebo. Patients and investigators were blind to the treatment throughout the duration of the study. At each visit, before, during, and after the treatment, clinical parameters of disease were assessed by interviewing the patients. These parameters included abdominal pain, general wellbeing, and number of bowel movements per day (BM); the relevant disease activity index (Lichtiger or CDAI) was finally calculated. Quality of life (QOL) was assessed by using the SF36 questionnaire (14, 17).

### Study Compounds and Treatments

#### UC Patients

Treatment was provided in the form of cigarettes. For the active arm, each cigarette contained 0.5 g of dried cannabis flowers, equivalent to 11.5 mg of THC. The cigarettes were comprised of dried flowers of genetically identical plants of *Cannabis sativa* var. Indica “Erez” (courtesy of Tikun Olam Ltd., Tel Aviv, Israel), known to contain 23% THC and <0.5% CBD. The placebo cigarettes contained cannabis flowers from which THC had been extracted as previously described (18). In short, dried flowers of *Cannabis sativa* var. Indica “Erez” (Tikun-Olam Ltd., Tel Aviv, Israel), known to contain 23% THC and <0.5% CBD and other cannabinoids, were soaked in 95% ethanol for 2 weeks. The procedure was repeated three times. Next, the flowers were covered with a mixture of herbal spirits and 0.025% *Saccharomyces cerevisiae* var. “18” (Courtesy Rimonest Ltd., Haifa, Israel) for three more days and then allowed to dry in the ambient air with ventilation for 72 h. The final product was tested for cannabinoids and shown to possess <0.4% THC with undetectable amounts of all other cannabinoids including CBD. All cigarettes were machine-made to ensure they looked identical.

#### CD Patients

Treatment was provided in the form of oil that was extracted from *Cannabis sativa* var. Indica “Avidekel” (courtesy of Tikun Olam Ltd., Tel Aviv, Israel). The composition of the oil was 8 mg/ml CBD and 2 mg/ml THC. Other cannabinoids were present in small quantities. The starting dose was one drop twice daily before meals, gradually raised until the patient felt a satisfactory effect (i.e., reduction in abdominal pain and diarrhea) or until side effects occurred. The maximal allowed dose was 20 drops/bid (i.e., 40 drops/day) with each drop being equivalent to 0.05 ml.

### Endocannabinoid Measurements

The extraction, purification, and quantification of serum eCBs were performed by stable isotope dilution liquid chromatography/tandem mass spectrometry (LC-MS/MS) as previously described (19). Briefly, total proteins were first precipitated using ice-cold acetone and Tris buffer (50 mM, pH 8.0). Next, samples were homogenized using a mixture of 0.5 ml ice-cold methanol/Tris buffer (50 mM, pH 8.0), 1:1, and 7 µl internal standard (22.4 ng d4-AEA). Following this, the homogenates were extracted using ice-cold CHCl_3_:MeOH (2:1, vol/vol) and then washed with ice-cold chloroform for three times. The samples were then dried under thin stream of nitrogen and reconstituted in MeOH. Analysis by LC-MS/MS was conducted on an AB Sciex (Framingham, MA, USA) Triple Quad 5500 Mass Spectrometer and a Shimadzu (Kyoto, Japan) ultrahigh performance liquid chromatography (UHPLC) System, while the liquid chromatographic separation was acquired *via* a Kinetex (Phenomenex) column (C18, 2.6 mm particle size, 100 × 2.1 mm). Sample levels of AEA, 2-AG, AA, PEA, and OEA were measured against a standard curve and then expressed as pmol/ml. Gradient elution mobile phases consisted of 0.1% formic acid in water (phase A) and 0.1% formic acid in acetonitrile (phase B). Gradient elution (250 μl/min) is described in **Table 1**.

**Table 1:** Gradient elution mobile phase.

Time (min)	Phase A, %	Phase B, %
0	50	50
3	50	50
10	30	70
15	25	75
15.5	10	90
20	10	90
21	50	50
28	50	50

### Immunohistochemistry

Biopsies were taken from inflamed sections in the colon. In UC, it was from the left colon, but in CD, it varied according to the inflammation in individual patients. Paraffin sections from biopsies taken before the clinical trial and/or 8 weeks later were deparaffinized in xylene and alcohol, rinsed in 1× phosphate-buffered saline (PBS), immersed in citrate buffer (pH 6.0), and microwaved for 15 min. Endogenous peroxidase activity was quenched in 3% H_2_O_2_. Samples were blocked using a goat serum and incubated with primary antibodies against FAAH and NAPE-PLD (**Table 2**) overnight. After washing, slides were incubated with horseradish-peroxidase-labeled polymer conjugated to a secondary antibody (**Table 2**), washed, and developed with AEC chromogen system (Covance Research Products, USA). Isotype-matched control antibodies were used to verify absence of nonspecific staining. Four and 10 patients were analyzed for NAPE-PLD and FAAH expression in the beginning and the end of the experiments in the cannabis and placebo groups, respectively.

**Table 2:** Antibody table.

Target	Source	Isotype	Company	Method
FAAH	Rabbit	Monoclonal IgG (Clone EPR7549)	Millipore	IHC, WB
NAPE-PLD	Rabbit	Polyclonal IgG	Novusbio	IHC, WB
Polymer detection kit (HRP) Mouse/Rabbit/Rat antibodies			Zytomed	IHC
GAPDH	Rabbit	IgG	Abcam	WB
Peroxidase conjugated antirabbit	Goat	Polyclonal IgG	Jackson ImmunoResearch Laboratories)	WB

### QuPath Digital Analysis

QuPath analysis is a software platform (https://qupath.github.io/) used for slide image analysis. Following staining with antibodies against FAAH and NAPE-PLD, we used QuPath analysis to evaluate FAAH staining in the photographed area, which contained both crypts and stromal cells. For both analyses, QuPath software was fixed to the following parameters: region identification-positive pixel count (Gaussian sigma, 2 µM; hematoxylin threshold, 0.1 OD; positive threshold, 0.2 OD (for FAAH) and 0.1 OD (for NAPE-PLD)]. The analyses were done on two photographs taken from two areas from each biopsy.

### Caco-2 Cell Growth

Caco-2 cells (colon carcinoma cells, kindly provided by Dr. Koltai, The Volcani Institute, Rishon-Lezion, Israel) were grown in Dulbecco’s modified Eagle’s medium (DMEM) media supplemented with 2 mM L-glutamine, antibiotics (50 µg/ml streptomycin, 100 IU/ml penicillin), and plasmocin (5 µg/ml).

### Cannabis Extraction

Cannabis plant samples were ground to a fine powder using an electrical grinder. Prior to phytocannabinoid extraction, several of the flowers underwent heat-decarboxylation in an oven at 120°C for 1 h. Approximately 5 g of the natural or decarboxylated flowers was accurately weighed and extracted with 50 ml ethanol. Samples were sonicated in an ultrasonic bath for 30 min and then agitated in an orbital shaker at 25°C for 15 min. Samples were then filtered under pressure through Whatman filter paper number 4, and the ethanol was evaporated under reduced pressure at 38°C using a rotary evaporator (Laborata 4000; Heidolph Instruments GmbH & Co. KG, Germany). For a more detailed description of the extraction and sample preparation, see Berman et al. (20).

### Exposure of Caco-2 Cells to Cannabis

Caco-2 cells were cultured with or without the two extracts of cannabis (Washed “Erez” with THC and Washed “Avidekel” without THC, 10 µg/ml) made by the Department of Biology, as described above. After 48 h incubation, the colon epithelial cells were harvested and their proteins extracted.

### Protein Extraction and Western Blotting

Caco-2 cells were washed with 1× PBS and lysed in buffer [as previously described (21)] on ice for 10 min. Protein lysate from 1 × 10^5^ cells was mixed (1:5) with loading buffer, denatured for 10 min at 65°C, separated on sodium dodecyl sulfate–polyacrylamide gel electrophoresis (SDS-PAGE), and transferred to a polyvinylidene-difluoride membrane. After blocking (5% dry milk), membranes were incubated at 4°C overnight with primary antibodies against FAAH, NAPE-PLD, and glyceraldehyde 3-phosphate dehydrogenase (GAPDH) (as described in **Table 2**). All antibodies were visualized using peroxidase-conjugated secondary antibody followed by enhanced chemiluminescence detection (Pierce, Rockford, IL, USA). Optical densities were visualized and measured as arbitrary units by LAS3000 Imager (Fujifilm). Results were normalized to GAPDH using Multi-gauge V3.0 program (Fujifilm).

### Statistics

Categorical variables were reported as number and percentage. Comparisons between baseline characteristics and eCBs level between first and third visits were evaluated by using Wilcoxon signed-rank test (https://www.socscistatistics.com/tests/signedranks/default2.aspx), and comparison between groups was evaluated using Mann–Whitney test (https://www.socscistatistics.com/tests/mannwhitney/default2.aspx).

A Student’s t-test using Excel software was used to analyze differences between Western blot results. An effect was considered significant when *p* < 0.05.

## Results

Thirteen patients with CD and nine patients with UC were treated with cannabis and 17 patients with CD and 10 with UC served as placebo groups. Clinical response of both UC and CD groups is described in **Tables 3** and **4**.

**Table 3:** Clinical parameters of UC patients.

UC	Number	Gender M/F %	Age	Lichtiger score		BM		QOL	
				Visit 1	Visit 3	Visit 1	Visit 3	Visit 1	Visit 3
Cannabis	9	4/5	40 ± 16	9.7 ± 1.2	4.9 ± 1.06*	3.9 ± 1.2	1.7 ± 0.6*	72.7 ± 6.7	98.2 ± 7.3*
Placebo	10	8/2	34 ± 9	11.6 ± 0.9	8.4 ± 0.9*^	5.4 ± 1.8	3.7 ± 1.36*	77.1 ± 3.7	82 ± 4.7^

Lichtiger score and BM and QOL levels in UC patients before and after cannabis/placebo use: UC patients were treated for 8 weeks with cannabis or placebo. At time 0 (visit 1) and 8 weeks following the cannabis or placebo use (visit 3), Lichtiger score, BM, and QOL levels were evaluated.

*Results significantly different from visit 1 (p < 0.05).

^Results significantly different between the placebo and the cannabis group (p < 0.05).

**Table 4:** Clinical parameters of CD patients.

CD	No.	Gender	Age	CDAI		BM		QOL	
		M/F		Visit 1	Visit 3	Visit 1	Visit 3	Visit 1	Visit 3
Cannabis	13	6/7	34 ± 15	278 ± 22	171 ± 30.7*	5.6 ± 0.78	3.7 ± 0.8*	75 ± 5.7	94.2 ± 5.2*
Placebo	17	8/9	35 ± 9	309 ± 27	236 ± 26.7*	6.8 ± 1.18	4.1 ± 0.8*	74.5 ± 4.48	72.5 ± 5.8^

CDAI score and BM and QOL levels in CD patients before and after cannabis/placebo use: CD patients were treated for 8 weeks with cannabis or placebo. At time 0 (visit 1) and 8 weeks following the cannabis or placebo use (visit 3), CDAI score, BM, and QOL levels were evaluated.

*Results significantly different from visit 1 (p < 0.05).

^Results significantly different between the placebo to the cannabis group (p < 0.05).

The levels of five eCBs (AEA, PEA, OEA, 2-AG, and AA) were assessed in the patients’ blood before and/or after 8 weeks of treatment (as described in **Table 5**).

**Table 5:** Number of UC and CD patients.

Treatment	UC			CD		
	Before	After	Paired results	Before	After	Paired results
**Cannabis**	9	8	8	11	13	10
**Placebo**	8	10	8	15	17	15

The number of UC and CD patients that participated in the study. Nineteen patients with UC and 30 patients with CD participated in the study. In some patients, sera were tested at the beginning and end of the experiment, while as in some patients, sera were sampled only in one-time point.

**Figure 1:**
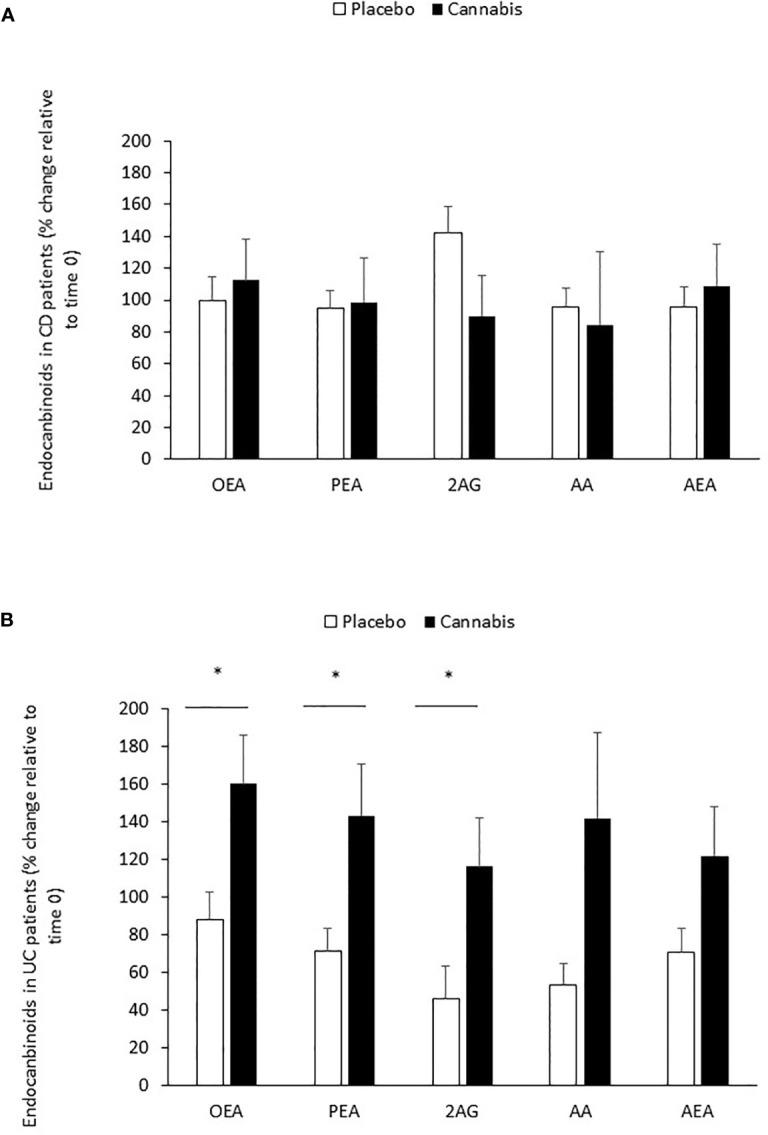
Changes in eCB levels during 8 weeks of cannabis or placebo use. **(A)** CD and **(B)** UC patients were treated for 8 weeks with cannabis or placebo. Before the beginning of cannabis/placebo use and 8 weeks later, blood was taken from the patients, and eCB levels were evaluated in the sera by using LC-MS/MS. The line represents eCB levels in time 0 (before cannabis use). *Results significantly different from time 0 (*p* < 0.05).

In CD patients (placebo and cannabis), the levels of all eCBs remained unaltered during the treatment period (**Table 6**; **Figure 1A**). However, in the placebo-treated UC patients, the levels of PEA, AEA, and AA decreased significantly after 8 weeks (**Table 6**). This reduction was prevented in cannabis-treated UC patients (**Table 6**). When the analysis was performed only on patients with data at the beginning and end of the experiment, we found that the percent change in the levels of OEA, PEA, and 2-AG between the two groups (placebo *vs.* cannabis) in UC patients was significantly different. A similar trend was also found in AA and AEA (**Figure 1B**). These results suggest that cannabis use can affect eCBs “tone” in UC patients.

**Table 6:** Effect of cannabis on eCB levels.

	OEA		PEA		2AG		AA		AEA	
	Before	After	Before	After	Before	After	Before	After	Before	After
**UC Cannabis**	16.3 ± 3.9	18.9 ± 2.35	4.2 ± 0.8	4.6 ± 0.5	10.6 ± 1.9	10.2 ± 2.7	3,806.7 ± 930	3,778.2 ± 607	460.5 ± 109	386.1 ± 62.8
**UC Placebo**	21.2 ± 2.3	16.9 ± 1.92	4.8 ± 0.2	3.2 ± 0.3*	13.6 ± 3.9	12.3 ± 3.9	4,552.6 ± 536	2,774.3 ± 411*	628.2 ± 62	422.9* ± 63.3
**CD Cannabis**	23.0 ± 3	22.8 ± 2.5	5.6 ± 0.7	5.1 ± 0.6	37.9 ± 7.5	32.6 ± 11.6	7,553.2 ± 1,785	4,943.1 ± 682	710.1 ± 94.3	737.1 ± 103
**CD Placebo**	27.3 ± 2.4	25.4 ± 3.3	5.1 ± 0.5	4.5 ± 0.5	37.9 ± 9.1	49.9 ± 12.7	7,957.7 ± 965	6,057 ± 661	822.3 ± 121	674.9 ± 59

The effect of cannabis and placebo treatment on eCB level in the sera of IBD patients. UC and CD patients were treated for 8 weeks with cannabis or placebo. One week before the beginning of the experiment and 8 weeks later, blood was taken from the patients, and eCB levels were evaluated in the sera by using LC-MS/MS. eCB levels are in pmol/ml.

*Levels of eCBs before and after cannabis/placebo use are significantly different, p < 0.05.

**Figure 2:**
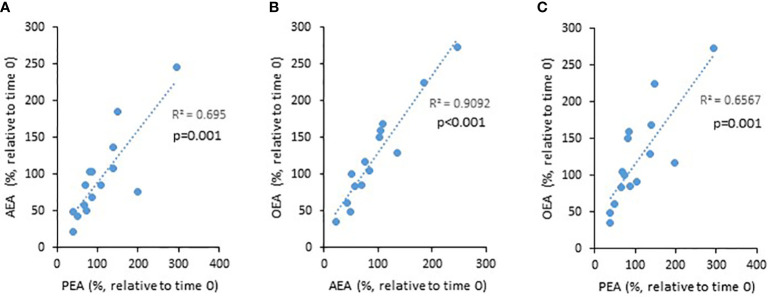
The relationship between the levels of eCBs throughout the 8 weeks trial. UC patients were treated for 8 weeks with cannabis or placebo. eCBs level were evaluated before the beginning of the experiment and 8 weeks later. The graphs describe the changes in eCBs level as a function of the changes in other eCBs level [**(A)** PEA vs. AEA, **(B)** AEA vs. OEA, and **(C)** PEA vs. OEA].

PEA, AEA, and OEA are produced by the enzyme NAPE-PLD, while 2-AG is produced by DAGL. Therefore, we next assessed correlations between the levels of PEA, AEA, and OEA, and 2-AG. Indeed, we found positive correlations between the NAPE-PLD-dependent eCBs (i.e., PEA *vs.* AEA, AEA *vs.* OEA, and PEA *vs.* OEA, **Figure 2**), but no correlation between these and 2-AG (data not shown). These results support the reliability of our tests.

**Figure 3:**
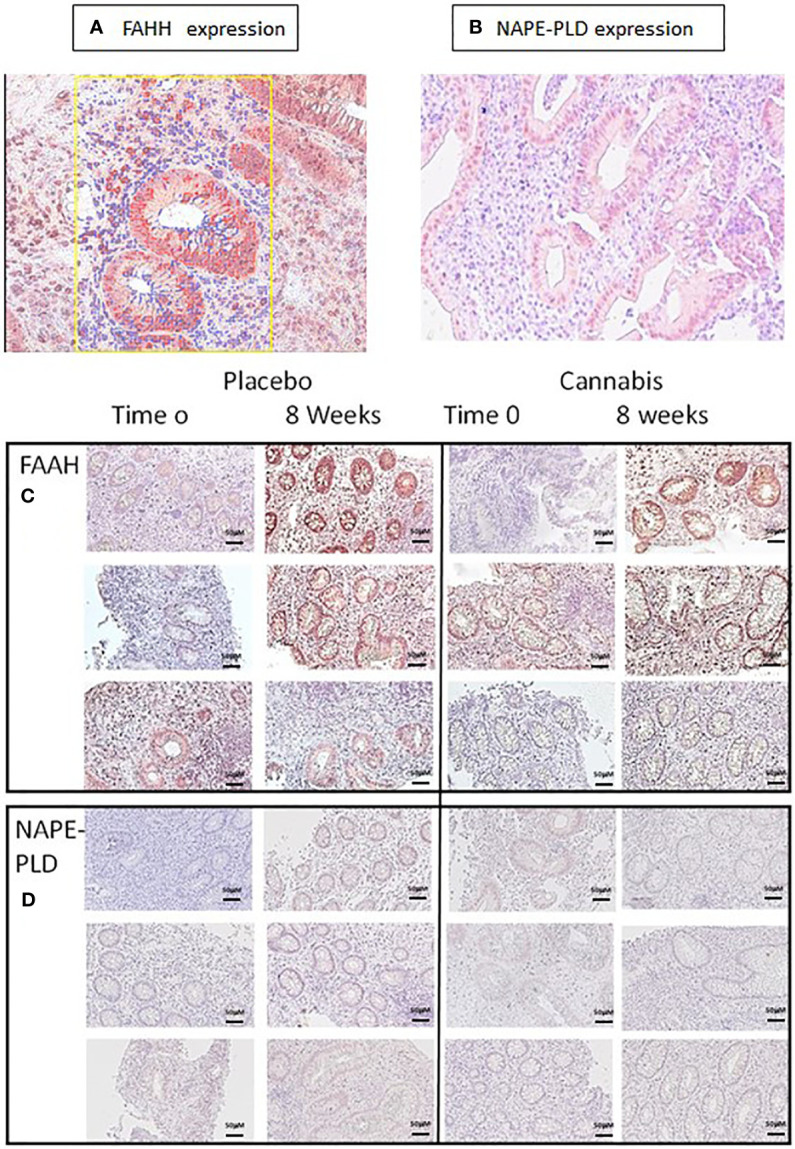
FAAH and NAPE-PLD expression in colon biopsies. **(A, C)** FAAH and **(B, D)** NAPE-PLD expression were evaluated in colon biopsies using immunohistochemistry. The expression of the proteins in the images was measured using QuPath digital analysis tool. Presented are representative pictures of colon biopsies stained with **(A)** anti-FAAH and **(B)** anti-NAPE-PLD. The yellow square in panel **(A)** illustrates a QuPath reading. The red markings indicate a positive staining for FAAH, while the blue ones indicate staining with hematoxylin. **(C, D)** Images of FAAH and NAPE-PLD staining from three patients treated with placebo or cannabis at the beginning of the experiment and 8 weeks later.

**Figure 4:**
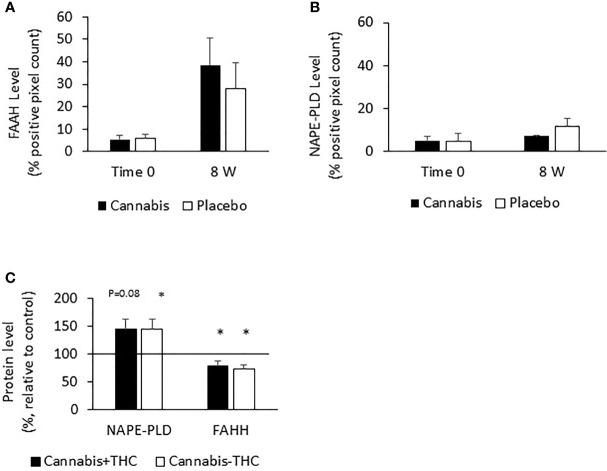
FAAH and NAPE-PLD expression levels in colon biopsies and Caco-2 cells treated with cannabis or controls. UC patients were treated for 8 weeks with cannabis or placebo. FAAH and NAPE-PLD levels were evaluated before the beginning of the experiment and 8 weeks later by using QuPath analysis digital tool. Panel **(A)** represents FAAH expression levels, and panel **(B)** represents NAPE-PLD expression levels. **(C)** Caco-2 cells were treated for 48 h with cannabis with/ without THC. The cells were then harvested and their proteins extracted and evaluated for FAAH and NAPE-PLD levels. **(A)** *Results significantly different from time 0; **(C)** *results significantly different from control.

The level of eCBs (PEA, AEA, and OEA) decreased throughout the study period in the UC placebo group. The enzyme FAAH degrades all these eCBs, while the enzyme NAPE-PLD participates in their synthesis. We assumed that increased levels of FAAH or decreased levels of NAPE-PLD may contribute to the reduced eCBs level found after 8 weeks in the UC placebo group and that cannabis consumption stabilized the levels of these enzymes and prevented the reduction in eCBs level. To test this hypothesis, we evaluated FAAH and NAPE-PLD levels in biopsies of UC patients before and after 8 weeks of treatment. We found that FAAH was expressed by the epithelial cells and by some of the stromal cells (**Figure 3A**), while NAPE-PLD was very weakly expressed by the epithelial cells of the crypt and to a greater extent in the apical surface of the epithelial border (**Figure 3B**). Very few cells in the stroma expressed NAPE-PLD. The expression of FAAH and NAPE-PLD varied in patients in both the placebo and cannabis groups, both at the beginning and at the end of the experiment (**Figures 3C, D**). We used QuPath digital software in order to evaluate FAAH and NAPE-PLD levels in the biopsies. FAAH levels increased over the 8 weeks of the study in both groups (from 6 and 4.6 to 19 and 24.6 in the placebo and cannabis groups, respectively; **Figures 3A** and **4A**). Combining the results from placebo and cannabis-treated patients showed that indeed the expression levels of FAAH significantly increased during the 8-week treatment period (*p* < 0.05). These findings may explain the decrease in eCBs level in UC placebo group but not the stable eCBs level found in the cannabis group. NAPE-PLD staining was weaker, and despite a slight increase in its levels, no significant differences were found between the beginnings of the experiment to its end and between the cannabis and placebo groups (**Figure 4B**). Combining the results from placebo and cannabis-treated patients, we could see a trend of increasing expression levels of NAPE-PLD during the 8 week treatment period (*p* = 0.1).

Since both enzymes were expressed by the epithelial cells, we also exposed Caco-2 (colon carcinoma cells) to two chemovars of cannabis, with and without THC for 48 h, extracted their proteins, and evaluated the effect of cannabis exposure on FAAH and NAPE-PLD expression. Similar to the biopsies results, we found that both cannabis extracts increased NAPE-PLD levels (placebo, *p <*0.05; cannabis, *p* = 0.08); yet, unlike the biopsy results, they reduced FAAH expression levels (*p* < 0.05, **Figure 4C**).

Our results suggest that the levels of the FAAH and NAPE-PLD enzymes in the two groups (cannabis and placebo) cannot explain the decrease in the level of eCBs in the placebo group, on the one hand, and the stability in their levels in the cannabis group, on the other hand, and that efforts will have to be made in the future to find the responsible mechanism.

**Figure 5:**
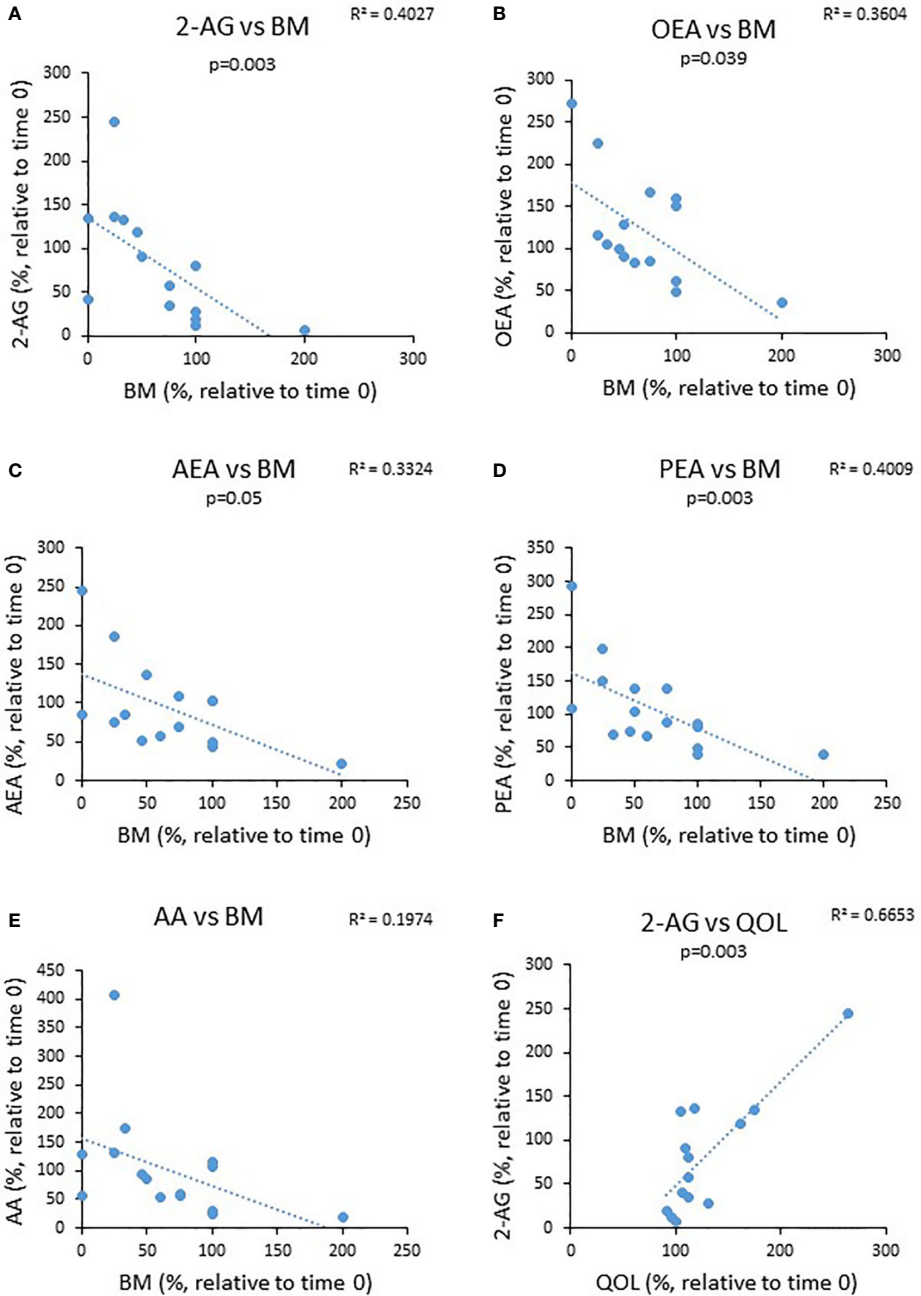
The relationship between changes in eCBs level during the trial to changes in clinical parameters. UC patients were treated for 8 weeks with cannabis or placebo. eCBs level, BM, QOL and Mayo score were evaluated before the beginning of the experiment and 8 weeks later. The graphs describe the changes in BM as a function ofthe changes in the different eCBs level **(A–E)** and **(F)** the change in QOL as a function of 2-AG level.

Next, we investigated a potential correlation between changes observed in bowel movements (BM) and QOL from the beginning to the end of the experiment and changes observed in eCBs level during this period. We found that the percent reduction in the levels of BM was negatively correlated with changes in the circulating AEA and OEA, whereas changes in the QOL were positively correlated with the levels of 2-AG (**Figure 5**). These results suggest that eCBs tone affects UC symptoms.

## Discussion

Many physiological factors, such as different diseases, exercise, sleep, and stress contribute to eCBs circulating levels. Previous studies even showed that the response of some eCBs to stress is different between men and women and, potentially, between races. Therefore, determination of eCBs at multiple time points may provide important information that cannot be obtained at a single time point (7).

Previous observations have shown that IBD patients have altered eCBs tone, but the effect of cannabis consumption on eCBs was not studied. The novelty of our study is that we tested the effect of cannabis on the patient’s eCBs blood concentration, by measuring eCBs level in the same patients at two time points, before and after treatment. Our self-controlled study design overcomes the variability between eCBs level in sera collected from different people and enables comparison of eCBs levels with clinical parameters, which were measured at the same time. We did not find significant changes in eCBs level in the sera of CD patients during cannabis treatment. However, the levels of PEA, AEA, and AA decreased significantly overtime in the placebo group of UC patients, while cannabis consumption prevented this decrease. The decrease in the level of eCBs in the UC placebo group surprised us because both groups had improvement in clinical condition. However, Lichtiger score and QOL were improved significantly more in the UC cannabis group compared to the UC placebo group. However, the fact that disease activity was significantly different between the groups at the end of the study is consistent with the changes observed in eCBs concentration and strengthens their reliability. It is also possible that some of the improvement that occurred in the placebo group was due to the placebo effect, as often happens (22).

Since changes in eCBs were observed only in UC patients, we continued our study only in this group. In an attempt to understand the different effects of cannabis and placebo on eCBs level, we continued to examine in patient’s biopsies the expression levels of FAAH and NAPE-PLD. We found that FAAH levels increased during the experimental period in the placebo and cannabis groups, and NAPE-PLD showed a trend toward elevation. These results could not explain why eCBs level was reduced in the placebo group but not in the cannabis group.

Previous studies reported changes in eCBs levels in IBD patients (23). Di Sabatino and colleagues showed that the content of AEA, but not of 2-AG and PEA, was significantly lower in inflamed versus uninflamed IBD mucosa, and this was paralleled by a low activity of NAPE-PLD and a high activity of FAAH (11). These findings are in line with the reduced AEA levels and increased FAAH expression that we found in the placebo group. In an additional study, circulating eCBs level was compared in IBD patients and healthy controls. AEA and OEA levels were found to be increased in UC and CD patients, while 2-AG levels were increased only in patients with CD (24). Despite the conflicting findings [(11, 24), our data], these results suggest that IBD can be associated with an altered eCBs “tone.”

To the best of our knowledge, this current study that followed cannabis-treated IBD patients over time is the first to find that cannabis alters eCBs “tone” and prevents the reduction in eCBs levels in UC. Previous studies showed that THC affects eCBs level. The administration of THC was previously shown to regulate eCBs level in mouse brains in an age- and region-dependent manner (25). In addition, treatment with THC increased NAPE-PLD transcription while it decreased FAAH expression levels after 24 h, but had the opposite effect after 72 h. Interestingly, AEA levels were increased after THC addition in that experimental model (26). Similarly, we found increased FAAH levels in the UC patients at the end of the experiment. This could explain the reduced eCBs found in the placebo group but not the stable eCBs level in the cannabis group. Exposure of Caco-2 cells to cannabis yielded similar results regarding the elevation in NAPE-PLD levels, yet opposite results regarding FAAH expression, which increased in the biopsies but decreased in the cells. This may reflect the fact that NAPE-PLD is expressed only by epithelial cells, whereas FAAH is expressed by both epithelial and stromal cells. In addition, while the biopsies reflect the biology of the entire organ, the *in vitro* system shows only the effect on epithelial cells. These results stress the need for a biological system that contains all cells of the organ such as a whole biopsy culture or 3D organoids.

Altogether, other studies in different diseases and models and our study demonstrate that cannabis alters the ECS and specifically eCBs “tone,” but the effect is different at different exposure times and in different cells.

Furthermore, our results suggest that another mechanism, which is not related to FAAH or NAPE-PLD levels, is responsible for the ability of cannabis treatment to prevent the reduction in eCBs levels. Additional enzymes involved in eCBs metabolism (10, 27) have been described, and it might be that their activities are affected by cannabis and responsible for the differences between the placebo and cannabis groups. This assumption is yet to be proved.

The altered eCBs level found in our study raised the question whether changes in eCBs level have relevance to the symptoms of UC. To try to answer this question, we tested the correlations of BM and QOL with the levels of each eCBs. Our study revealed a positive correlation between alterations in circulating 2-AG levels of UC patients and QOL and negative correlations between changes in PEA, AEA, and OEA levels of these patients to BM. In other words, a higher eCBs level was correlated with fewer bowel movements and a better QOL.

Indeed, bowel movements are highly affected by GI motility and gut permeability, which are known to be modulated by the ECS (9, 28). Specifically, AEA and 2-AG have been found to modulate epithelial cell layer permeability (28), and AEA, PEA, and OEA have been found to reduce intestinal motility in mice (10, 29). This is in line with the distribution of CBRs in neurons of the myenteric and submucosal plexuses. These previous observations further support our results, which demonstrated that cannabis treatment resulting in stable eCBs level reduces the number of BM.

Another important question is raised by the differences observed in our study between patients with CD and those with UC. A previous study that analyzed mRNA levels of ECS-related genes (synthesizing and degrading enzymes and cannabinoid receptors) found that intestinal mucosal biopsies from UC and CD patients showed different patterns of gene expression between the two and between those of healthy people (24). Interestingly, while in UC patients there was an improvement in the endoscopic activity in the cannabis treated patients, and to a lesser degree in the placebo group, no endoscopic improvement was observed in the CD group (14, 15). The fact that the differences in eCBs level were observed only in the group with endoscopic improvement reinforced our results. Nevertheless, it should be taken into account that the two patient groups (UC and CD) were treated with two different cannabis preparations (THC-rich cigarettes and CBD-rich oil), and it is possible that the differences in treatment rather than the difference in the disease were responsible for the different effect of cannabis on the level of endocannabinoids.

The strength of our study is the availability of both clinical data and blood samples before and after cannabis use (8 weeks). Eight weeks of cannabis use are long enough for cannabinoids to accumulate in adipose tissues and reach a steady state, thus truly reflecting their effect on the ECS (30). The weaknesses are the small group of patients. Another weakness of the study is that the two groups of patients, UC and CD, were treated with different cannabis preparations. Yet, we find great interest in the fact that the results of two different cannabis preparations came out different.

In conclusion, our study suggests that cannabis use may affect eCBs tone in UC patients. This affect has a beneficial effect on UC symptoms.

## Data Availability Statement

The raw data supporting the conclusions of this article will be made available by the authors, without undue reservation.

## Ethics Statement

The studies involving human participants were reviewed and approved by Ethics committee of Meir Hospital, Kfar Saba, Israel. The patients/participants provided their written informed consent to participate in this study.

## Author Contributions

ST devised the study, did the immunohistochemistry and Western blotting, and participated in writing the manuscript. SA did the serum endocannabinoid analysis. DM did the preparation and extraction of cannabis and helped writing the manuscript. RH did the serum endocannabinoid analysis. AN did the serum endocannabinoid analysis. NA did the immunohistochemistry, measurement of tissue endocannabinoid levels, and Caco-2 experiment. FK participated in devising the study and writing the manuscript, and participated in the clinical cannabis studies. LD participated in immunohistochemistry, measurement of tissue endocannabinoid levels, and Caco-2 experiment. JT did the serum endocannabinoid analysis and participated in writing the manuscript. TN devised and carried out the clinical cannabis studies, devised the current study, and participated in writing the manuscript. All authors contributed to the article and approved the submitted version.

## Funding

FK was supported, in part, by the Josefina Maus and Gabriela Cesarman Chair for Research in Liver Diseases, Sackler Faculty of Medicine, Tel Aviv University.

## Conflict of Interest

The authors declare that the research was conducted in the absence of any commercial or financial relationships that could be construed as a potential conflict of interest.

## Publisher’s Note

All claims expressed in this article are solely those of the authors and do not necessarily represent those of their affiliated organizations, or those of the publisher, the editors and the reviewers. Any product that may be evaluated in this article, or claim that may be made by its manufacturer, is not guaranteed or endorsed by the publisher.

## References

[1] Katsanos KH Papadakis KA. Inflammatory Bowel Disease: Updates on Molecular Targets for Biologics. Gut Liver (2017) 11(4):455–63. 10.5009/gnl16308 PMC5491079 28486793

[2] da Silva BC Lyra AC Rocha R Santana GO. Epidemiology, Demographic Characteristics and Prognostic Predictors of Ulcerative Colitis. World J Gastroenterol (2014) 20(28):9458–67. 10.3748/wjg.v20.i28.9458 PMC4110577 25071340

[3] Ungar B Kopylov U. Advances in the Development of New Biologics in Inflammatory Bowel Disease. Ann Gastroenterol (2016) 29(3):243–8. 10.20524/aog.2016.0027 PMC4923809 27366024

[4] Weiss A Friedenberg F. Patterns of Cannabis Use in Patients With Inflammatory Bowel Disease: A Population Based Analysis. Drug Alcohol Depend (2015) 156:84–9. 10.1016/j.drugalcdep.2015.08.035 26422462

[5] Hasenoehrl C Storr M Schicho R. Cannabinoids for Treating Inflammatory Bowel Diseases: Where are We and Where do We Go? Expert Rev Gastroenterol Hepatol (2017) 11(4):329–37. 10.1080/17474124.2017.1292851 PMC5388177 28276820

[6] Alhouayek M Muccioli GG. The Endocannabinoid System in Inflammatory Bowel Diseases: From Pathophysiology to Therapeutic Opportunity. Trends Mol Med (2012) 18(10):615–25. 10.1016/j.molmed.2012.07.009 22917662

[7] Hillard CJ. Circulating Endocannabinoids: From Whence Do They Come and Where are They Going? Neuropsychopharmacology (2018) 43(1):155–72. 10.1038/npp.2017.130 PMC5719092 28653665

[8] Toczek M Malinowska B. Enhanced Endocannabinoid Tone as a Potential Target of Pharmacotherapy. Life Sci (2018) 204:20–45. 10.1016/j.lfs.2018.04.054 29729263

[9] Acharya N Penukonda S Shcheglova T Hagymasi AT Basu S Srivastava PK. Endocannabinoid System Acts as a Regulator of Immune Homeostasis in the Gut. Proc Natl Acad Sci USA (2017) 114(19):5005–10. 10.1073/pnas.1612177114 PMC5441729 28439004

[10] Ambrose T Simmons A. Cannabis, Cannabinoids, and the Endocannabinoid System-Is There Therapeutic Potential for Inflammatory Bowel Disease? J Crohns Colitis (2019) 13(4):525–35. 10.1093/ecco-jcc/jjy185 PMC6441301 30418525

[11] Di Sabatino A Battista N Biancheri P Rapino C Rovedatti L Astarita G. The Endogenous Cannabinoid System in the Gut of Patients With Inflammatory Bowel Disease. Mucosal Immunol (2011) 4(5):574–83. 10.1038/mi.2011.18 21471961

[12] Nallathambi R Mazuz M Ion A Selvaraj G Weininger S Fridlender M. Anti-Inflammatory Activity in Colon Models Is Derived From Δ9-Tetrahydrocannabinolic Acid That Interacts With Additional Compounds in Cannabis Extracts. Cannabis Cannabinoid Res (2017) 2(1):167–82. 10.1089/can.2017.0027 PMC5627671 29082314

[13] Ambrose T Simmons A. Cannabis, Cannabinoids and the Endocannabinoid System - is There Therapeutic Potential for Inflammatory Bowel Disease? J Crohns Colitis (2018). 10.1093/ecco-jcc/jjy185 PMC6441301 30418525

[14] Naftali T Bar-Lev Schleider L Scklerovsky Benjaminov F Konikoff FM Matalon ST Ringel Y. Cannabis is Associated With Clinical But Not Endoscopic Remission in Ulcerative Colitis: A Randomized Controlled Trial. PloS One (2021) 16(2):e0246871. 10.1371/journal.pone.0246871 33571293 PMC7877751

[15] Naftali T Bar-Lev Schleider L Almog S Meiri D Konikoff FM. Oral CBD-Rich Cannabis Induces Clinical But Not Endoscopic Response in Patients With Crohn’s Disease, a Randomized Controlled Trial. J Crohns Colitis (2021) 15:jjab069. 10.1093/ecco-jcc/jjab069 33858011

[16] Altman DG Bland JM. How to Randomise. BMJ (1999) 319(7211):703–4. 10.1136/bmj.319.7211.703 PMC1116549 10480833

[17] Naftali T Bar-Lev Schlieder L Konikoff F Ringel Y. Cannabis Induces Clinical Response But No Endoscopic Response in Crohn’s Disease Patients. UEG (2018) 6(8_suppl).

[18] Naftali T Schlieder LBL Benjaminov FS Lish I Hirsh J Konikoff FM. Sa1744-Cannabis Induces Clinical and Endoscopic Improvement in Moderately Active Ulcerative Colitis. Gastroenterolog (2018) 154(6):S-378. 10.1093/ecco-jcc/jjx180.525

[19] Azar S Sherf-Dagan S Nemirovski A Webb M Raziel A Keidar A. Circulating Endocannabinoids Are Reduced Following Bariatric Surgery and Associated with Improved Metabolic Homeostasis in Humans. Obes Surg (2019) 29(1):268–76. 10.1007/s11695-018-3517-0 30244333

[20] Berman P Futoran K Lewitus GM Mukha D Benami M Shlomi T. A New ESI-LC/MS Approach for Comprehensive Metabolic Profiling of Phytocannabinoids in Cannabis. Sci Rep (2018) 8(1):14280. 10.1038/s41598-018-32651-4 30250104 PMC6155167

[21] Epstein Shochet G Drucker L Pomeranz M Fishman A Pasmanik-Chor M Tartakover-Matalon S. First Trimester Human Placenta Prevents Breast Cancer Cell Attachment to the Matrix: The Role of Extracellular Matrix. Mol Carcinog (2017) 56(1):62–74. 10.1002/mc.22473 26859229

[22] Enck P Klosterhalfen S. Placebos and the Placebo Effect in Drug Trials. Handb Exp Pharmacol (2019) 260:399–431. 10.1007/164_2019_269 31463606

[23] Diab J Al-Mahdi R Gouveia-Figueira S Hansen T Jensen E Goll R. A Quantitative Analysis of Colonic Mucosal Oxylipins and Endocannabinoids in Treatment-Naive and Deep Remission Ulcerative Colitis Patients and the Potential Link With Cytokine Gene Expression. Inflamm Bowel Dis (2019) 25(3):490–7. 10.1093/ibd/izy349 PMC6383859 30476077

[24] Grill M Högenauer C Blesl A Haybaeck J Golob-Schwarzl N Ferreirós N. Members of the Endocannabinoid System are Distinctly Regulated in Inflammatory Bowel Disease and Colorectal Cancer. Sci Rep (2019) 9(1):2358. 10.1038/s41598-019-38865-4 30787385 PMC6382821

[25] Leishman E Murphy M Mackie K Bradshaw HB. Delta(9)-Tetrahydrocannabinol Changes the Brain Lipidome and Transcriptome Differentially in the Adolescent and the Adult. Biochim Biophys Acta Mol Cell Biol Lipids (2018) 1863(5):479–92. 10.1016/j.bbalip.2018.02.001 PMC5987162 29408467

[26] Maia J Midão L Cunha SC Almada M Fonseca BM Braga J. Effects of Cannabis Tetrahydrocannabinol on Endocannabinoid Homeostasis in Human Placenta. Arch Toxicol (2019) 93(3):649–58. 10.1007/s00204-019-02389-7 30659320

[27] Blankman JL Simon GM Cravatt BF. A Comprehensive Profile of Brain Enzymes That Hydrolyze the Endocannabinoid 2-Arachidonoylglycerol. Chem Biol (2007) 14(12):1347–56. 10.1016/j.chembiol.2007.11.006 PMC2692834 18096503

[28] Pesce M D'Alessandro A Borrelli O Gigli S Seguella L Cuomo R. Endocannabinoid-Related Compounds in Gastrointestinal Diseases. J Cell Mol Med (2018) 22(2):706–15. 10.1111/jcmm.13359 PMC5783846 28990365

[29] Bashashati M Storr MA Nikas SP Wood JT Godlewski G Liu J. Inhibiting Fatty Acid Amide Hydrolase Normalizes Endotoxin-Induced Enhanced Gastrointestinal Motility in Mice. Br J Pharmacol (2012) 165(5):1556–71. 10.1111/j.1476-5381.2011.01644.x PMC3372737 21883147

[30] Lucas CJ Galettis P Schneider J. The Pharmacokinetics and the Pharmacodynamics of Cannabinoids. Br J Clin Pharmacol (2018) 84(11):2477–82. 10.1111/bcp.13710 PMC6177698 30001569

